# Severity of Airflow Obstruction Based on FEV_1_/FVC Versus FEV_1 _Percent Predicted in the General U.S. Population

**DOI:** 10.1164/rccm.202310-1773OC

**Published:** 2024-04-10

**Authors:** Helena Backman, Lowie E. G. W. Vanfleteren, David M. Mannino, Magnus Ekström

**Affiliations:** ^1^ Department of Public Health and Clinical Medicine, Section for Sustainable Health, the OLIN Unit, Umeå University, Umea, Sweden;; ^2^ COPD Center, Department of Pulmonary Medicine and Allergology, Sahlgrenska University Hospital, Gothenburg, Sweden;; ^3^ Department of Internal Medicine and Clinical Nutrition, Institute of Medicine, Sahlgrenska Academy, University of Gothenburg, Gothenburg, Sweden;; ^4^ Department of Medicine, University of Kentucky College of Medicine, Lexington, Kentucky;; ^5^ COPD Foundation, Washington, D.C.; and; ^6^ Faculty of Medicine, Department of Clinical Sciences Lund, Respiratory Medicine, Allergology, and Palliative Medicine, Lund University, Lund, Sweden

**Keywords:** chronic obstructive pulmonary disease, airflow limitation, airway obstruction, prognosis

## Abstract

**Rationale:** According to the Global Initiative for Obstructive Lung Disease (GOLD), the FEV_1_/FVC ratio is used to confirm the presence of airflow obstruction in the diagnosis of chronic obstructive pulmonary disease (COPD), whereas FEV_1_ percent predicted normal value (FEV_1_%pred) is used for grading its severity. The STaging of Airflow obstruction by the FEV_1_/FVC Ratio (STAR), and its prediction of adverse outcomes, has not been evaluated in general populations.

**Objectives:** To compare the STAR (FEV_1_/FVC) and the GOLD (FEV_1_%pred) classifications for the severity of airflow limitation in terms of exertional breathlessness and mortality in the general U.S. population.

**Methods:** Severity stages according to the STAR and GOLD were applied to the multiethnic National Health and Nutrition Examination Survey of 2007–2012, including people ages 18–80 years, using a postbronchodilatory FEV_1_/FVC ratio of <0.70 to define airflow obstruction in both staging systems. Prevalence of the severity stages STAR 1–4 and GOLD 1–4 was calculated, and associations with breathlessness and mortality were analyzed by multinomial logistic regression and Cox regression, respectively.

**Measurements and Main Results:** STAR versus GOLD severity staging of airflow obstruction showed similar associations with breathlessness and all-cause mortality, regardless of ethnicity and/or race. In those with airflow obstruction, the correlation between the two classification systems was 0.461 (*P* < 0.001). STAR reclassified 59% of GOLD 2 subjects as having mild airflow obstruction (STAR 1). Compared with GOLD 1, STAR 1 was more clearly differentiated from nonobstruction in terms of both breathlessness and mortality.

**Conclusions:** FEV_1_/FVC and FEV_1_%pred as measures of airflow limitation severity show similar predictions of breathlessness and mortality in the adult U.S. population across ethnicity groups. However, Stage 1 differed more clearly from nonobstruction on the basis of FEV_1_/FVC ratio than FEV_1_%pred.

**At a Glance Commentary**

**Scientific Knowledge on the Subject**

According to the Global Initiative for Obstructive Lung Disease (GOLD), the FEV_1_/FVC ratio is used to confirm airflow obstruction in chronic obstructive pulmonary disease diagnosis, whereas FEV_1_ percent predicted normal value (FEV_1_%pred) is used for severity grading. The STaging of Airflow obstruction by the FEV_1_/FVC Ratio (STAR) was recently shown to discriminate mortality similarly compared with GOLD, but with a more uniform gradation of severity in a selected cohort of smokers and ex-smokers.

**What This Study Adds to the Field**

In this study of the general adult U.S. population, FEV_1_/FVC and FEV_1_%pred, as measures of severity of airflow limitation, showed similar prediction of breathlessness and all-cause mortality, regardless of ethnicity and/or race, indicating that both low FEV_1_/FVC and low FEV_1_%pred are important predictors of symptoms as well as mortality. Although the two classification systems were correlated, STAR Stage 1 was more clearly differentiated from nonobstruction compared with GOLD Stage 1 in terms of both breathlessness and mortality. This was related to a substantial proportion of GOLD Stage 2 subjects who were reclassified as STAR Stage 1.

Chronic obstructive pulmonary disease (COPD) is defined as a heterogeneous lung condition characterized by chronic respiratory symptoms that are due to abnormalities of the airways and/or alveoli that cause persistent and often progressive airflow obstruction (1). According to the Global Initiative for Obstructive Lung Disease (GOLD), the diagnosis of COPD is confirmed by spirometry showing airflow obstruction, defined as a postbronchodilator FEV_1_/FVC ratio below 0.70 (1). After the diagnosis is made, GOLD currently classifies the severity of airflow obstruction on the basis of the impairment in FEV_1_ but not expressed as proportionate to the FVC, but rather in reference to percent predicted normal value (FEV_1_%pred) based on the distribution of FEV_1_ in a healthy nonsmoking population. Indeed, FEV_1_%pred strongly predicts survival in people with COPD (2). However, FEV1 is highly related to FVC in both obstructive and non-obstructive populations (3). Hence, a lower FEV_1_%pred in COPD can reflect restrictive spirometry that is potentially related to heart conditions, musculoskeletal conditions, obesity, or other pathology (4). In addition, current FEV_1_ grading is dependent on accurate and updated reference values, which are not available in many settings. From the viewpoint of the severity of obstruction, it is logical to identify groups with more severe obstruction on the basis of the ratio used to diagnose the disease (i.e., FEV_1_/FVC) (3). In addition, the FEV_1_/FVC ratio has been shown to be more stable across race and/or ethnicity groups (5–8).

Recently proposed by Bhatt and colleagues (9), STAR (the STaging of Airflow obstruction by the FEV_1_/FVC Ratio) provided further evidence for such an approach in large patient datasets, the COPDGene study (*n* = 10,132), and two cohorts studied in Pittsburgh (*n* = 2,017). Airflow obstruction classified by STAR versus GOLD showed similar discrimination of mortality, but STAR classification resulted in a more uniform gradation of disease severity that differentiated patients’ symptoms, disease burden, and prognosis better than the GOLD classification (9). Patients with mild airflow obstruction were better differentiated from those without airflow obstruction. Nevertheless, some questions remain. The COPDGene study only included former and current smokers ages 45–80 years, and further validation is needed in a general multiethnic adult population that includes younger adults and nonsmokers with high-quality standardized spirometry (10).

Therefore, we aimed to compare the STAR classification (measured with FEV_1_/FVC) with the GOLD classification (measured with FEV_1_%pred) for grading the severity of airflow limitation in terms of exertional breathlessness and mortality in the general U.S. population.

## Methods

### Study Design and Population

This was a population-based analysis of noninstitutionalized adults in the United States using data from the National Health and Nutrition Examination Survey (NHANES) of 2007–2012 (11–13). We included people ages 18 to 80 years old, with data on measured height and spirometry. Participants provided written consent to participate in the NHANES using a protocol approved by the National Center for Health Statistics Research Ethics Review Board (11–13). All the data used in the present analysis were deidentified, were publicly available, and did not need additional ethical approval by the Swedish Ethical Review Authority in accordance with national research regulations. The study is reported in accordance with Strengthening the Reporting of Observational Studies in Epidemiology guidelines (14).

### Assessments

Data on age, sex, and self-reported smoking status (never, former, or current), and race and/or ethnicity (Mexican American, other Hispanic, White, Black, or other) were gathered from personal interviews. Measured weight (in kilograms), height (in centimeters), and spirometry data were obtained from mobile examination centers. Dynamic spirometry was performed in accordance with guidelines from the American Thoracic Society and the European Respiratory Society (15). Bronchodilation was performed in individuals with signs of obstruction, and a total of *n* = 997 (7% of the subjects) underwent bronchodilation testing. Values were recorded as the highest obtained value (pre- or postbronchodilator), and normal values for the FEV_1_ and FVC were predicted using the Global Lung Function Initiative Global reference equations (5).

STAR categories were defined in accordance with Bhatt and colleagues (9) as follows: Stage 1 (FEV_1_/FVC ⩾0.60 to <0.70), Stage 2 (FEV_1_/FVC ⩾0.50 to <0.60), Stage 3 (FEV_1_/FVC ⩾0.4 to <0.50), and Stage 4 (FEV_1_/FVC <0.40). GOLD stages were defined as a FEV_1_/FVC <0.7 and a FEV_1_%pred of: ⩾80% (Stage 1), ⩾50% to <80% (Stage 2), ⩾30% to <50% (Stage 3), or <30% (Stage 4) (16). Similarly for STAR and GOLD, people with a FEV_1_/FVC ⩾0.70 were categorized as Stage 0, and Stages 3 and 4 were merged because of low numbers in the population sample.

Outcomes data on exertional breathlessness (“shortness of breath either when hurrying on the level or walking up a slight hill,” corresponding to a modified Medical Research Council score ⩾1) were available for people ages 40 and older, and all-cause mortality was longitudinally assessed using standardized NHANES procedures through December 31, 2019 (7).

### Statistical Analyses

The study population was weighted (using published NHANES weights for people undergoing examinations including spirometry) to represent the noninstitutionalized U.S. population during the 6-year period. For all analyses, variance estimates were produced using Taylor series linearization methods (17), as recommended for NHANES.

Descriptive data were tabulated for the study population using mean (SD) for normally distributed continuous variables and frequency (percentage) for categorical variables. FEV_1_/FVC was plotted against FEV_1_%pred to evaluate the distribution of the STAR and GOLD stages. The correlation between the classifications was analyzed using Kendall’s τ_B_ (ranging from −1 to 1, where −1 = perfect negative correlation, 0 = no correlation, and 1 = perfect positive correlation). Reclassification of people from GOLD to STAR stages was evaluated using cross-tabulation and a Sankey diagram.

As the GOLD and STAR categorizations were applied to the same people, the comparative analyses were independent of (adjusted for) participant characteristics by design. Associations with breathlessness were analyzed as relative risk ratios using multinomial logistic regression models, and associations with mortality were evaluated using Kaplan-Meier plots and as hazard ratios using Cox regression. Goodness of fit was assessed using the Hosmer-Lemeshow test. Estimates were reported both unadjusted as well as adjusted for age, sex, and body mass index (BMI, calculated as units by kg/m^2^), with 95% confidence intervals. The predictive discrimination for each model was analyzed using percentage of outcomes correctly classified for breathlessness and the C statistic for mortality. Additionally, the predictive discrimination was calculated separately for each race and/or ethnicity group and sex.

The associations with mortality were also visualized using cubic splines (18) for FEV_1_/FVC and FEV_1_%pred as continuous variables, centered around the respective median values as reference. Splines with three knots were used, as more knots did not improve model fit evaluated using Akaike’s information criterion and the Bayesian information criterion.

Furthermore, additional analyses were performed by splitting subjects without airflow obstruction into two groups on the basis of having an FEV_1_ less than the lower limit of normal (LLN) or an FEV_1_ equal to or greater than the LLN.

Statistical analyses were performed with Stata, Version 17.0 (StataCorp LP). This study adheres to the guidelines of Transparent Reporting of a Multivariable Prediction Model for Individual Prognosis or Diagnosis (or, TRIPOD) (19).

## Results

A total of 14,123 (50% women) subjects were included (Table 1), representing 186,930,379 people when weighted to the adult U.S. population. Participants had a mean age of 45 years (SD = 17) and a mean BMI of 28.9 (SD = 6.8), with 5,134 (36%) having a BMI ⩾30; 7,299 (52%) of participants were never-smokers, 2,999 (21%) were former smokers, and 3,030 (21%) were current smokers. There were 2,291 Mexican Americans, 1,529 identified as other Hispanic, 5,928 Whites, 3,130 Blacks, and 1,245 identified as other race and/or ethnicity (*see* Table E1 in the online supplement).

**Table 1.** Participant Characteristics

Characteristics	Men	Women	All
*n*	7,093	7,030	14,123
Female, *n* (%)	0 (0)	7,030 (100)	7,030 (50.3)
Age, mean (SD)	44.9 (17.2)	45.3 (17.0)	45.1 (17.1)
Weight, kg, mean (SD)	87.2 (20.5)	76.1 (20.7)	81.7 (21.4)
Height, cm, mean (SD)	174.7 (7.7)	161.2 (7.1)	168.0 (10.1)
Body mass index, kg/m^2^, mean (SD)	28.5 (6.1)	29.3 (7.5)	28.9 (6.8)
*n* (%)			
<18.5	84 (1.0)	154 (2.4)	238 (1.7)
18.5 to <25	2,009 (27.5)	2,091 (33.4)	4,100 (30.4)
25 to <30	2,645 (38.4)	2,006 (28.6)	4,651 (33.5)
⩾30	2,355 (33.1)	2,779 (35.6)	5,134 (34.4)
Smoking status, *n* (%)			
Never	3,085 (47.0)	4,214 (58.9)	7,299 (53.0)
Former	1,793 (25.1)	1,206 (19.8)	2,999 (22.4)
Current	1,772 (23.6)	1,258 (18.1)	3,030 (20.9)
Missing	443 (4.2)	352 (3.2)	795 (3.7)
Asthma, *n* (%)	392 (5.7)	657 (9.3)	1,049 (7.5)
Diabetes mellitus, *n* (%)	717 (7.4)	690 (7.1)	1,407 (7.2)
Hypertension, *n* (%)	2,090 (26.9)	2,143 (26.6)	4,233 (26.7)
Heart failure, *n* (%)	150 (1.6)	100 (1.2)	250 (1.4)
Ischemic heart disease, *n* (%)	380 (4.3)	172 (1.8)	552 (3.1)
FEV_1_/FVC, mean (SD)	0.8 (0.1)	0.8 (0.1)	0.8 (0.1)
FEV_1_/FVC < 0.70, *n* (%)	980 (13.2)	541 (8.5)	1,521 (10.8)
FEV_1_, L, mean (SD)	3.6 (0.9)	2.6 (0.7)	3.1 (0.9)
FEV_1_%pred, mean (SD)	97.9 (16.7)	98.1 (16.7)	98.0 (16.7)
FVC (L), mean (SD)	4.6 (1.0)	3.3 (0.7)	3.9 (1.1)
STAR stage, *n* (%)			
0	6,113 (86.8)	6,489 (91.5)	12,602 (89.2)
1	748 (10.3)	411 (6.6)	1,159 (8.4)
2	155 (1.9)	96 (1.4)	251 (1.6)
3	55 (0.9)	28 (0.4)	83 (0.6)
4	22 (0.2)	6 (0.1)	28 (0.2)
GOLD stage, *n* (%)			
0	6,113 (86.8)	6,489 (91.5)	12,602 (89.2)
1	574 (8.6)	256 (4.5)	830 (6.6)
2	355 (4.1)	237 (3.4)	592 (3.7)
3	49 (0.5)	46 (0.5)	95 (0.5)
4	2 (0.0)	2 (0.0)	4 (<0.1)
Breathlessness, mMRC score ⩾1, only available for ages ⩾40 yr, *n* (%)	1,062 (24.9)	1,491 (32.8)	2,553 (29.0)
Deaths, *n* (%)	668 (7.2)	432 (4.6)	1,100 (5.9)
Follow-up time in years, median (interquartile range)	9.8 (8.2–11.2)	9.8 (8.3–11.2)	9.8 (8.3–11.2)

*Definition of abbreviations*: GOLD = Global Initiative for Chronic Obstructive Lung Disease; mMRC = modified Medical Research Council Breathlessness scale; %pred = percent predicted normal value; STAR = STaging of Airflow obstruction by the FEV_1_/FVC Ratio.

Comorbid conditions represent self-reported diagnosed conditions. Asthma refers to current asthma (still have asthma). Ischemic heart disease refers to self-reported history of coronary artery disease or myocardial infarction. Data are presented as frequencies (population-weighted percentages), unless otherwise specified. Breathlessness data were available and analyzed for people ages 40 and older. Data were weighted against the U.S. population. The numbers represent body mass index, kg/m^2^, categories, *n* (%).

**Figure 1.**
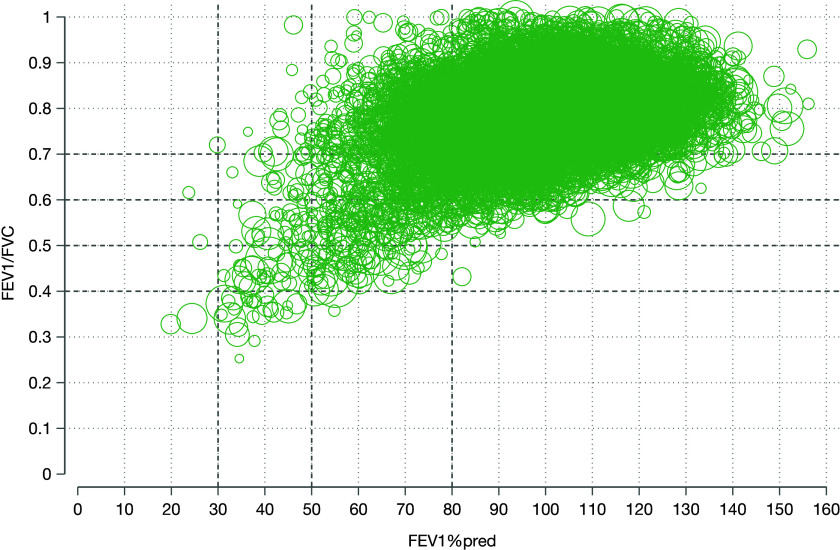
Plot of FEV_1_/FVC against FEV_1_ percent predicted normal value (FEV_1_%pred) according to the Global Lung Function Initiative Global reference values, illustrating distributions of the STaging of Airflow obstruction by the FEV_1_/FVC Ratio and Global Initiative for Obstructive Lung Disease severity stages in the U.S. population. The size of the circles represents the number of individuals with similar values, with larger circles indicating larger numbers.

**Figure 2.**
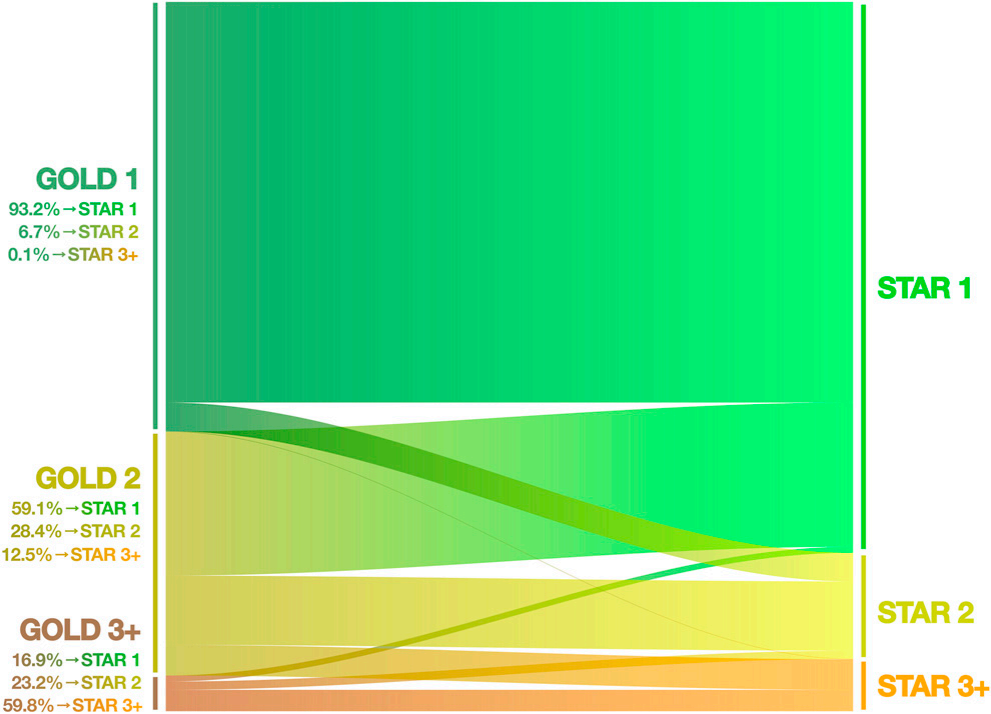
Reclassification of subjects from Global Initiative for Obstructive Lung Disease (GOLD) to STaging of Airflow obstruction by the FEV_1_/FVC Ratio (STAR) stage. The figure illustrates the percentage of reclassification from each GOLD stage to the different STAR stages. The height of the bands is proportional to the number of individuals among those with airflow obstruction.

FEV_1_/FVC is plotted against FEV_1_%pred in Figure 1. In total, 12,602 (89.2%) individuals had no airflow obstruction. Regarding the STAR classification, 1,159 (8.2%), 251 (1.8%), 83 (0.6%), and 28 (0.2%) subjects were STAR Stages 1–4. The corresponding distributions of GOLD Stages 1–4 were 830 (5.9%), 592 (4.2%), 95 (0.7%), and 4 (<0.1%). Among those with airflow obstruction (FEV_1_/FVC <0.7; i.e., Stages 1–4), the STAR and GOLD classifications were moderately correlated (Kendall’s τ_B_, 0.461, *P* < 0.001). The largest redistributions were from GOLD Stage 2 to STAR 1 (59.1%) and to STAR 3+ (12.5%), and from GOLD Stage 3+ to STAR 2 (23.7%) (Figure 2). Of people in GOLD Stage 1, 93.2% remained in STAR 1, and 6.7% were reclassified to STAR 2.

**Figure 3.**
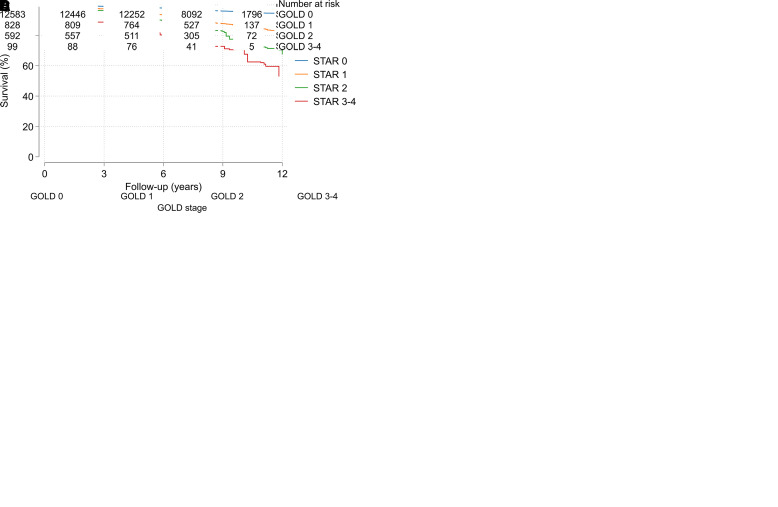
Breathlessness and mortality by STaging of Airflow obstruction by the FEV_1_/FVC Ratio (STAR) and Global Initiative for Obstructive Lung Disease (GOLD) stage. (*A* and *B*) The probability of breathlessness (with 95% confidence interval) is illustrated by (*A*) STAR category and (B) GOLD category. (*C* and *D*) Mortality in terms of Kaplan-Meier survival curves based on weighted data, along with the unweighted numbers at risk in the study sample, is illustrated by (*C*) STAR category and (*D*) GOLD category.

**Figure 4.**
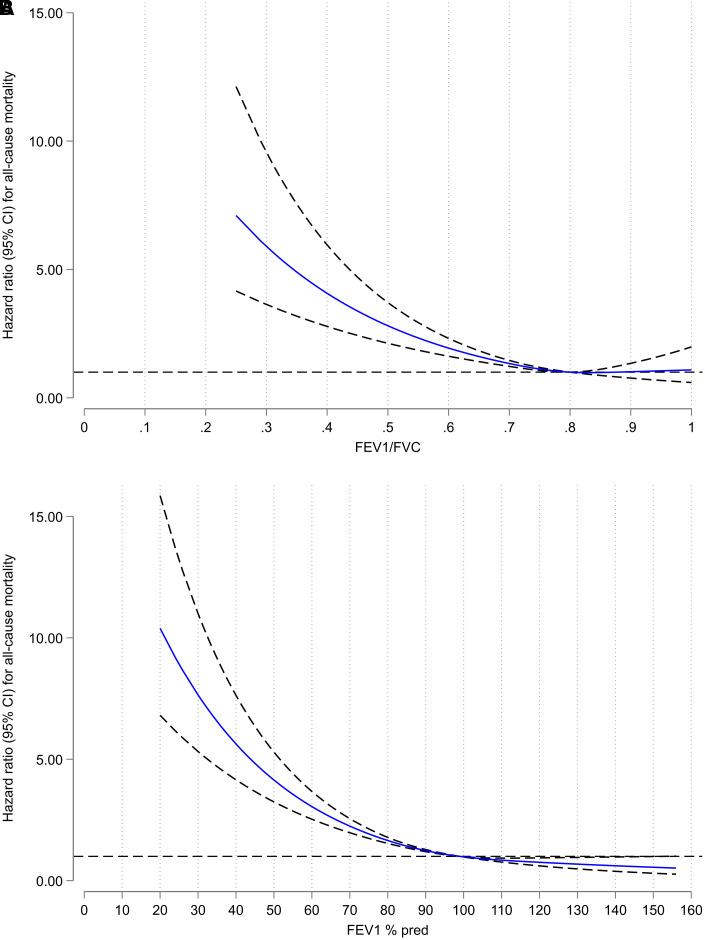
Mortality by (*A*) FEV_1_/FVC and (*B*) FEV_1 _percent predicted normal value (FEV_1_%pred) in the U.S. population. Hazard ratios with 95% confidence intervals (CI) were calculated by Cox regression using cubic splines with three knots for FEV_1_/FVC and FEV_1_%pred as continuous variables in two separate models, both with all-cause mortality as outcome and age, sex, and body mass index as covariates.

Regarding both breathlessness (available for subjects ages 40 and older) and mortality (available for all subjects ages 18 and older), the risk increased by increasing STAR and GOLD stages (Figure 3). As expected, the subpopulation ages 40 and older had a slightly higher prevalence of obstruction (*see* Table E2). Notably, in all subjects, STAR 1 discriminated both breathlessness and mortality significantly from people without obstruction, whereas these discriminations were less robust for GOLD Stage 1 (Figure 3). Aside from the differences seen related to STAR and GOLD Stage 1, there was a similar overall discriminative ability for STAR and GOLD in predicting breathlessness and mortality, unadjusted and adjusted for age, sex, and BMI (Tables 2 and E3). The proportions correctly classified in the adjusted breathlessness models were 71% for both STAR and GOLD, and the C-statistics for the adjusted mortality models were 0.81 for both STAR and GOLD (Table 2). The prediction was similar for STAR and GOLD across race and/or ethnicity groups (Table 3) and sex (*see* Table E4). When looking at people with obstruction (FEV_1_/FVC <0.7; i.e., Stages 1–4) only, the proportions correctly classified in the breathlessness models were 63% for both FEV_1_/FVC and FEV_1_%pred when included as continuous variables in the models, and the C-statistic for the mortality models was 0.695 for FEV_1_/FVC and 0.712 for FEV_1_%pred. Also, the shapes of associations between mortality and the FEV_1_/FVC and FEV_1_%pred were similar when these spirometry indices were analyzed as continuous variables (using splines) among all, in separate models adjusted for age, sex, and BMI (Figure 4).

**Table 2.** Breathlessness and Mortality by STAR and GOLD Stage

Stage	Breathlessness Relative Risk Ratio (95% CI)		Mortality Hazard Ratio (95% CI)	
	Crude	Adjusted*	Crude	Adjusted*
STAR				
0	1	1	1	1
1	1.6 (1.2–2.0)	1.8 (1.4–2.4)	3.2 (2.6–3.9)	1.4 (1.2–1.7)
2	3.2 (2.2–4.8)	4.1 (2.8–6.0)	5.8 (4.2–8.0)	2.0 (1.4–2.9)
3–4	8.9 (4.8–16.5)	12.5 (6.2–25.2)	9.1 (5.8–15.2)	3.5 (2.3–5.3)
—	Correctly classified = 70%	Correctly classified = 71%	C statistic = 0.61 (95% CI, 0.60–0.62)	C statistic = 0.81 (95% CI, 0.79–0.82)
GOLD				
0	1	1	1	1
1	1.1 (0.8–1.5)	1.3 (1.0–1.8)	2.8 (2.3–3.5)	1.2 (0.9–1.5)
2	4.3 (3.3–5.8)	4.9 (3.6–6.6)	5.0 (4.0–6.2)	2.1 (1.7–2.6)
3–4	8.1 (4.6–14.4)	9.7 (4.9–19.2)	12.9 (7.9–20.9)	5.3 (3.5–8.2)
—	Correctly classified = 70%	Correctly classified = 71%	C statistic = 0.61 (95% CI, 0.60–0.63)	C statistic = 0.81 (95% CI, 0.80–0.82)

*Definitions of abbreviations*: CI = confidence interval; GOLD = Global Initiative for Chronic Obstructive Lung Disease; STAR = STaging of Airflow obstruction by the FEV_1_/FVC Ratio.

Hosmer-Lemeshow *P* values for the adjusted breathlessness models were 0.092 for models including classification by STAR and 0.061 for models including classification by GOLD.

* Adjusted for age, sex, and body mass index.

**Table 3.** Prediction of Breathlessness and Mortality Using FEV_1_/FVC or FEV_1_%pred by Race and/or Ethnicity

Variable	Mexican American	Other Hispanic	White	Black	Other	All
*N*	2,291	1,529	5,928	3,130	1,245	14,123
Breathlessness, % correctly classified						
FEV_1_/FVC	75	73	70	69	82	71
FEV_1_%pred	75	73	71	70	81	71
Mortality, C statistic (95% CI)						
FEV_1_/FVC	0.81 (0.79–0.82)	0.80 (0.79–0.82)	0.81 (0.79–0.82)	0.81 (0.79–0.82)	0.80 (0.79–0.82)	0.81 (0.79–0.82)
FEV_1_%pred	0.82 (0.81–0.83)	0.82 (0.80–0.83)	0.82 (0.81–0.83)	0.82 (0.80–0.83)	0.82 (0.80–0.83)	0.82 (0.81–0.83)

*Definition of abbreviations*: CI = confidence interval; %pred = percent predicted normal value.

Breathlessness and mortality were analyzed using multinomial logistic regression and Cox regression, respectively. FEV_1_/FVC and FEV_1_%pred are included as continuous variables in the models. All models were adjusted for age, sex, and body mass index.

The additional analyses in which subjects without obstruction with an FEV_1_ equal to or greater than the LLN were used as the reference category, instead of all subjects without obstruction, confirmed a clearer separation of STAR 1 than of GOLD 1 from the reference category (*see* Table E5).

## Discussion

The main findings of this population-based study are that the STAR and GOLD severity gradings of airflow obstruction showed similar prediction of breathlessness and all-cause mortality among the general U.S. population, ages 18–80 years, regardless of ethnicity and/or race. The patterns of association with these two adverse outcomes were similar, indicating that both a low FEV_1_/FVC and low FEV_1_%pred are important predictors of symptoms as well as mortality. However, although the two classification systems were correlated, STAR 1 differed more clearly in terms of breathlessness and mortality from people without obstruction compared with GOLD Stage 1, mainly because of a reclassification of a large proportion of GOLD Stage 2 subjects into STAR Stage 1.

These findings extend those of Bhatt and colleagues (9) by providing a multiethnic U.S. general population perspective, which includes nonsmokers and younger adults, all with high-quality standardized spirometry. Our findings indicated an overall similar discrimination regarding mortality for the STAR and GOLD classifications of airflow obstruction in the general population, in line with the results shown in ever-smokers in COPDGene and the two Pittsburgh cohorts (9). Furthermore, our results confirmed that STAR resulted in a better differentiation than GOLD between people with mild airflow obstruction and people without airflow obstruction in the general U.S. population. These findings also align with those of a similar prediction of mortality measured with FEV_1_/FVC versus FEV_1_%pred in general populations using data from the Burden of Obstructive Lung Disease study (20).

From a general population perspective, it is important to notice that the STAR reclassifies a large proportion of moderately obstructed subjects according to GOLD (Stage 2) to mild obstruction according to STAR (Stage 1). Indeed, STAR classifies the severity of obstruction accounting for FVC. FEV_1_ and FVC are acknowledged as markers of general health and survival, which probably should be taken into account in a complex syndrome like COPD, which is commonly seen in a multimorbid context (21). Thus, whether it is clinically appropriate to narrow the COPD severity grading exclusively to the level of obstruction is a matter of discussion. After all, current treatment recommendations today are based on expressed symptoms, history of exacerbations, and biomarkers and not on degree of airflow obstruction (1).

An important argument for the STAR approach lies in the fact that it is based simply on observed FEV_1_/FVC ratio instead of a reliance on normative reference equations. This seems appealing, especially in populations with lack of validated or regularly updated spirometry equations (22). It has been argued that the choice of reference equations is currently the major source of uncertainty and that we need a more uniform interpretation of spirometry results (23). It should be emphasized that the crude observed FEV_1_/FVC ratio is more stable than FEV_1_%pred across different populations and ethnic groups. Here, we used the Global Lung Function Initiative Global equations to avoid bias due to race adjustments in the comparisons between STAR and GOLD grading and found similar discrimination for mortality and breathlessness for STAR and GOLD within each of the race and/or ethnicity categories available in NHANES.

Regardless of staging system, subjects without airflow obstruction are defined as having a FEV_1_/FVC ratio of ⩾0.70, which might include subjects with preserved ratio impaired spirometry (PRISm). These subjects may have increased mortality compared with those with normal lung function (24). It is interesting that, when we divided subjects without obstruction according to the presence or absence of PRISm, indeed, we found higher mortality among those with PRISm than among those without PRISm, which emphasizes the need for better understanding of early COPD. Furthermore, we could also confirm that STAR 1 differentiated both breathlessness and mortality more clearly than GOLD did in subjects who had neither airflow obstruction nor PRISm.

In recent years, the limitations of using FEV_1_/FVC as the sole diagnostic criterion has been recognized, and the importance of other lung physiological, structural, and functional abnormalities has been highlighted to identify subjects at risk (25–27). A classification of severity that is based on the FEV_1_/FVC ratio might be considered the severity of the specific trait “airflow obstruction” in the broader context of COPD. Notably, for grading airflow obstruction, this approach of severity classification performs at least equally well, compared with the current GOLD classification, in terms of determining breathlessness and mortality in the general population.

Strengths of the present study include the population-based design and well-characterized cohort including data on race and/or ethnicity, also enabling a long-term follow-up of mortality. The cohort contains a large group of subjects without airflow limitation for comparison of the severity stages, thus enabling differentiation also for mild obstruction compared with nonobstruction. Quality control of spirometry was performed. Our study limitations include the small proportion with GOLD Stage 4, thus requiring the merging of Stages 3 and 4, and studies including more individuals with severe airflow obstruction would be valuable. Furthermore, as bronchodilation was only given to participants with signs of obstruction in NHANES, our study is mainly based on prebronchodilator values. This is also a weakness of the reference equations, which are solely based on prebronchodilatory spirometry values.

A clinical implication of our findings is that STAR provides a simple method to both identify and evaluate the severity of airflow obstruction using one single metric, the FEV_1_/FVC ratio, without the need to relate FEV_1_ to a predicted normal value, which might not be representative for the underlying population. Thus, it could be clinically useful in many settings, including in primary care and lower resource settings, and might simplify and facilitate the improved use and interpretation of spirometry. Of note, regardless of the choice of severity staging system, spirometry findings should always be evaluated in the wider clinical context, and most clinicians would not act on spirometry findings alone without any symptoms and radiographic or other clinical changes. Finally, we would like to propose that STAR should be evaluated in different populations in different countries and welcome more research and increased understanding of early COPD before we make any major changes in COPD recommendations.

In conclusion, FEV_1_/FVC and FEV_1_%pred as measures of severity of airflow limitation predict mortality and breathlessness similarly in the multiethnic adult U.S. population. However, Stage 1 is differentiated from nonobstruction more clearly when it is based on FEV_1_/FVC than on FEV_1_%pred. From a population perspective, severity staging that is based on FEV_1_/FVC as proposed by STAR as compared with FEV_1_%pred as proposed by GOLD would, on average, shift individuals with airflow obstruction into milder severity stages.

## Supplemental Materials

10.1164/rccm.202310-1773OC Online Data Supplement

## References

[1] Bøtker MT Stengaard C Andersen MS Søndergaard HM Dodt KK Niemann T *et al.* Dyspnea, a high-risk symptom in patients suspected of myocardial infarction in the ambulance? A population-based follow-up study *Scand J Trauma Resusc Emerg Med* 2016 24 15 26872739 10.1186/s13049-016-0204-9 PMC4751637

[2] Anthonisen NR Wright EC Hodgkin JE Prognosis in chronic obstructive pulmonary disease *Am Rev Respir Dis* 1986 133 14 20 3510578 10.1164/arrd.1986.133.1.14

[3] Burney P Coming off the GOLD standard *Lancet Respir Med* 2014 2 174 176 24621678 10.1016/S2213-2600(14)70040-2

[4] Kulbacka-Ortiz K Triest FJJ Franssen FME Wouters EFM Studnicka M Vollmer WM *et al.* Restricted spirometry and cardiometabolic comorbidities: results from the international population based BOLD study *Respir Res* 2022 23 34 35177082 10.1186/s12931-022-01939-5 PMC8855577

[5] Bowerman C Bhakta NR Brazzale D Cooper BR Cooper J Gochicoa-Rangel L *et al.* A race-neutral approach to the interpretation of lung function measurements *Am J Respir Crit Care Med* 2023 207 768 774 36383197 10.1164/rccm.202205-0963OC

[6] Moffett AT Bowerman C Stanojevic S Eneanya ND Halpern SD Weissman GE Global, race-neutral reference equations and pulmonary function test interpretation *JAMA Netw Open* 2023 6 e2316174 37261830 10.1001/jamanetworkopen.2023.16174 PMC10236239

[7] Ekström M Backman H Mannino D Clinical implications of the Global Lung Function Initiative race-neutral spirometry reference equations in terms of breathlessness and mortality *Am J Respir Crit Care Med* 2024 209 104 106 37187171 10.1164/rccm.202212-2229LE

[8] Ekström M Mannino D Research race-specific reference values and lung function impairment, breathlessness and prognosis: analysis of NHANES 2007–2012 *Respir Res* 2022 23 271 36182912 10.1186/s12931-022-02194-4 PMC9526909

[9] Bhatt SP Nakhmani A Fortis S Strand MJ Silverman EK Sciurba FC *et al.* FEV_1_/FVC severity stages for chronic obstructive pulmonary disease *Am J Respir Crit Care Med* 2023 208 676 684 37339502 10.1164/rccm.202303-0450OC PMC10515563

[10] Calverley PMA A star is born: a new approach to assessing chronic obstructive pulmonary disease severity *Am J Respir Crit Care Med* 2023 208 647 648 37486264 10.1164/rccm.202306-1106ED PMC10515562

[11] National Center for Health Statistics Atlanta Centers for Disease Control and Prevention 2007 https://wwwn.cdc.gov/nchs/nhanes/continuousnhanes/manuals.aspx?BeginYear=2007

[12] National Center for Health Statistics Atlanta Centers for Disease Control and Prevention 2009 https://wwwn.cdc.gov/nchs/nhanes/continuousnhanes/default.aspx?BeginYear=2009

[13] National Center for Health Statistics Atlanta Centers for Disease Control and Prevention 2011 https://wwwn.cdc.gov/nchs/nhanes/continuousnhanes/default.aspx?BeginYear=2011

[14] von Elm E Altman DG Egger M Pocock SJ Gøtzsche PC Vandenbroucke JP STROBE Initiative Strengthening the Reporting of Observational Studies in Epidemiology (STROBE) statement: guidelines for reporting observational studies *BMJ* 2007 335 806 808 17947786 10.1136/bmj.39335.541782.AD PMC2034723

[15] Miller MR Hankinson J Brusasco V Burgos F Casaburi R Coates A *et al.* ATS/ERS Task Force Standardisation of spirometry *Eur Respir J* 2005 26 319 338 16055882 10.1183/09031936.05.00034805

[16] Global strategy for prevention, diagnosis and management of COPD: 2023 report Deer Park, IL Global Initiative for Chronic Obstructive Lung Disease 2023 https://goldcopd.org/2023-gold-report-2/

[17] Lavrakas P Encyclopedia of survey research methods Thousand Oaks, CA Sage 2008

[18] Durrleman S Simon R Flexible regression models with cubic splines *Stat Med* 1989 8 551 561 2657958 10.1002/sim.4780080504

[19] Collins GS Reitsma JB Altman DG Moons KG Transparent reporting of a multivariable prediction model for individual prognosis or diagnosis (TRIPOD): the TRIPOD statement *BMJ* 2015 350 g7594 25569120 10.1136/bmj.g7594

[20] Coton S Vollmer WM Bateman E Marks GB Tan W Mejza F *et al.* Burden of Obstructive Lung Disease Study investigators Severity of airflow obstruction in chronic obstructive pulmonary disease (COPD): proposal for a new classification *COPD* 2017 14 469 475 28799856 10.1080/15412555.2017.1339681

[21] Fabbri LM Celli BR Agustí A Criner GJ Dransfield MT Divo M *et al.* COPD and multimorbidity: recognising and addressing a syndemic occurrence *Lancet Respir Med* 2023 11 1020 1034 37696283 10.1016/S2213-2600(23)00261-8

[22] Allinson JP Afzal S Çolak Y Jarvis D Backman H van den Berge M *et al.* CADSET Clinical Research Collaboration Changes in lung function in European adults born between 1884 and 1996 and implications for the diagnosis of lung disease: a cross-sectional analysis of ten population-based studies *Lancet Respir Med* 2022 10 83 94 34619103 10.1016/S2213-2600(21)00313-1

[23] Brusasco V Pellegrino R Pulmonary function interpretative strategies: from statistics to clinical practice *Eur Respir J* 2022 60 2200317 35835474 10.1183/13993003.00317-2022

[24] Wan ES Balte P Schwartz JE Bhatt SP Cassano PA Couper D *et al.* Association between preserved ratio impaired spirometry and clinical outcomes in us adults *JAMA* 2021 326 2287 2298 34905031 10.1001/jama.2021.20939 PMC8672237

[25] Stolz D Mkorombindo T Schumann DM Agusti A Ash SY Bafadhel M *et al.* Towards the elimination of chronic obstructive pulmonary disease: a Lancet Commission *Lancet* 2022 400 921 972 36075255 10.1016/S0140-6736(22)01273-9 PMC11260396

[26] Lowe KE Regan EA Anzueto A Austin E Austin JHM Beaty TH *et al.* COPDGene^®^ 2019: redefining the diagnosis of chronic obstructive pulmonary disease *Chronic Obstr Pulm Dis (Miami)* 2019 6 384 399 31710793

[27] Celli B Fabbri L Criner G Martinez FJ Mannino D Vogelmeier C *et al.* Definition and nomenclature of chronic obstructive pulmonary disease: time for its revision *Am J Respir Crit Care Med* 2022 206 1317 1325 35914087 10.1164/rccm.202204-0671PP PMC9746870

