# Supplementary material for: Severity of Airflow Obstruction Based on FEV1/FVC Versus FEV1 Percent Predicted in the General U.S. Population
Source: Am J Respir Crit Care Med. 2024 Apr 10;210(11):1308–16. doi: 10.1164/rccm.202310-1773OC (PMC11622431; doi:10.1164/rccm.202310-1773OC)
Supplement: Online Data Supplement [file rccm.202310-1773OCS1.docx]

**Severity of Airflow Obstruction Based on FEV_1_/FVC vs FEV_1_% of Predicted in the General US Population**

Helena Backman, PhD, Lowie E.G.W. Vanfleteren, MD, PhD, David M. Mannino, MD, Magnus Ekström, MD, PhD

**ONLINE DATA SUPPLEMENT**

**Table S1.** Characteristics of participants by race/ethnicity.

| **Characteristics** | Mexican American | Other Hispanic | White | Black | Other |
| --- | --- | --- | --- | --- | --- |
| N | 2,291 | 1,529 | 5,928 | 3,130 | 1,245 |
| Female | 1,119 (48.8%) | 823 (53.8%) | 2,915 (49.2%) | 1,569 (50.1%) | 604 (48.5%) |
| Age, mean (SD) | 42.1 (16.1) | 44.8 (16.7) | 47.0 (17.3) | 45.3 (17.4) | 41.7 (16.0) |
| Weight (kg), mean (SD) | 78.8 (18.5) | 77.5 (18.8) | 83.2 (21.3) | 87.4 (23.5) | 70.4 (17.9) |
| Height (cm), mean (SD) | 163.4 (9.6) | 163.9 (9.7) | 170.4 (9.7) | 169.8 (9.5) | 165.7 (9.5) |
| Body mass index (kg/m^2^), mean (SD) | 29.5 (6.0) | 28.8 (6.2) | 28.6 (6.8) | 30.3 (7.8) | 25.5 (5.5) |
| <18.5 | 17 (0.7%) | 18 (1.2%) | 108 (1.8%) | 52 (1.7%) | 43 (3.5%) |
| 18.5 to <25 | 479 (20.9%) | 387 (25.3%) | 1,815 (30.6%) | 766 (24.5%) | 653 (52.4%) |
| 25 to <30 | 879 (38.4%) | 581 (38.0%) | 1,952 (32.9%) | 890 (28.4%) | 349 (28.0%) |
| ≥30 | 916 (40.0%) | 543 (35.5%) | 2,053 (34.6%) | 1,422 (45.4%) | 200 (16.1%) |
| Smoking status |  |  |  |  |  |
| Never | 1,311 (62.2%) | 876 (61.0%) | 2,686 (47.1%) | 1,625 (55.8%) | 801 (68.2%) |
| Former | 431 (20.4%) | 301 (21.0%) | 1,554 (27.3%) | 528 (18.1%) | 185 (15.7%) |
| Current | 366 (17.4%) | 259 (18.0%) | 1,458 (25.6%) | 758 (26.0%) | 189 (16.1%) |
| Asthma | 83 (3.6%) | 110 (7.2%) | 500 (8.4%) | 284 (9.1%) | 72 (5.8%) |
| Diabetes mellitus | 238 (10.4%) | 153 (10.0%) | 475 (8.0%) | 447 (14.3%) | 94 (7.6%) |
| Hypertension | 516 (22.5%) | 415 (27.1%) | 1,790 (30.2%) | 1,249 (39.9%) | 263 (21.1%) |
| Heart failure | 22 (1.0%) | 18 (1.2%) | 119 (2.0%) | 79 (2.5%) | 12 (1.0%) |
| Ischemic heart disease | 65 (2.8%) | 47 (3.1%) | 301 (5.1%) | 107 (3.4%) | 32 (2.6%) |
| FEV_1_/FVC, mean (SD) | 0.81 (0.06) | 0.80 (0.07) | 0.77 (0.09) | 0.79 (0.08) | 0.81 (0.08) |
| FEV_1_/FVC < 0.70 | 108 (4.7%) | 101 (6.6%) | 904 (15.2%) | 322 (10.3%) | 86 (6.9%) |
| FEV_1_ (L), mean (SD) | 3.19 (0.84) | 3.03 (0.87) | 3.25 (0.95) | 2.81 (0.84) | 3.00 (0.88) |
| FEV_1_%pred, mean (SD) | 105.8 (14.0) | 101.9 (15.3) | 100.4 (16.2) | 86.9 (15.1) | 95.1 (15.0) |
| FVC (L), mean (SD) | 3.95 (1.00) | 3.76 (1.03) | 4.21 (1.13) | 3.53 (0.98) | 3.69 (1.02) |
| STAR stage |  |  |  |  |  |
| 0 | 2,183 (95.3%) | 1,428 (93.4%) | 5,024 (84.8%) | 2,808 (89.7%) | 1,159 (93.1%) |
| 1 | 96 (4.2%) | 81 (5.3%) | 674 (11.4%) | 241 (7.7%) | 67 (5.4%) |
| 2 | 8 (0.3%) | 15 (1.0%) | 159 (2.7%) | 55 (1.8%) | 14 (1.1%) |
| 3 | 3 (0.1%) | 4 (0.3%) | 56 (0.9%) | 18 (0.6%) | 2 (0.2%) |
| 4 | 1 (0.0%) | 1 (0.1%) | 15 (0.3%) | 8 (0.3%) | 3 (0.2%) |
| GOLD stage |  |  |  |  |  |
| 0 | 2,183 (95.3%) | 1,428 (93.4%) | 5,024 (84.8%) | 2,808 (89.7%) | 1,159 (93.1%) |
| 1 | 82 (0.6%) | 60 (3.9%) | 558 (9.4%) | 88 (2.8%) | 42 (3.4%) |
| 2 | 23 (1.0%) | 35 (2.3%) | 301 (5.1%) | 199 (6.4%) | 34 (2.7%) |
| 3 | 3 (0.1%) | 6 (0.4%) | 44 (0.7%) | 33 (1.1%) | 9 (0.7%) |
| 4 | 0 (0.0%) | 0 (0.0%) | 1 (0.0%) | 2 (0.1%) | 1 (0.1%) |
| Breathlessness (mMRC ≥ 1) | 312 (25.7%) | 253 (27.8%) | 1,234 (33.6%) | 619 (32.7%) | 135 (21.5%) |
| Deaths | 98 (4.3%) | 89 (5.8%) | 595 (10.1%) | 274 (8.8%) | 44 (3.5%) |

Data are presented as frequency (percentage) unless otherwise specified. Breathlessness data were available and analyzed for people aged ≥40 years. Data were weighted against the US population. *Abbreviations:* FEV_1_ = forced expired volume in one second; FVC = forced vital capacity; GOLD = Global Initiative for Chronic Obstructive Lung Disease; mMRC = modified Medical Research Council breathlessness scale; pred = predicted normal value; SD = standard deviation; STAR = Staging of Airflow Obstruction by Ratio.

**Table S2.** Characteristics of all participants and for participants aged 40 years or older.

| **Characteristics** | **All** | **All aged ≥40y** |
| --- | --- | --- |
| N | 14,123 | 8,234 |
| Female | 7,030 (50.3%) | 4,197 (50.4%) |
| Age, mean (SD) | 45.1 (17.1) | 57.1 (10.8) |
| Weight (kg), mean (SD) | 81.7 (21.4) | 82.8 (20.5) |
| Height (cm), mean (SD) | 168.0 (10.1) | 167.2 (10.1) |
| Body mass index (kg/m^2^), mean (SD) | 28.9 (6.8) | 29.5 (6.6) |
| <18.5 | 238 (1.7%) | 92 (1.1%) |
| 18.5 to <25 | 4,100 (30.4%) | 1,922 (23.1%) |
| 25 to <30 | 4,651 (33.5%) | 2,967 (35.6%) |
| ≥30 | 5,134 (34.4%) | 3,343 (40.2%) |
| Smoking status |  |  |
| Never | 7,299 (53.0%) | 4,238 (50.9%) |
| Former | 2,999 (22.4%) | 2,394 (28.8%) |
| Current | 3,030 (20.9%) | 1,687 (20.3%) |
| Missing | 795 (3.7%) | 5 (0.1%) |
| Asthma | 1,049 (7.5%) | 591 (7.1%) |
| Diabetes mellitus | 1,407 (7.2%) | 1,296 (15.6%) |
| Hypertension | 4,233 (26.7%) | 3,643 (43.8%) |
| Heart failure | 250 (1.4%) | 236 (2.8%) |
| Ischemic heart disease | 552 (3.1%) | 529 (6.4%) |
| FEV_1_/FVC, mean (SD) | 0.8 (0.1) | 0.8 (0.1) |
| FEV_1_/FVC < 0.70 | 1,521 (10.8%) | 1,374 (16.5%) |
| FEV_1_ (L), mean (SD) | 3.1 (0.9) | 2.7 (0.8) |
| FEV_1_%pred, mean (SD) | 98.0 (16.7) | 95.9 (18.1) |
| FVC (L), mean (SD) | 3.9 (1.1) | 3.6 (1.0) |
| STAR stage |  |  |
| 0 | 12,602 (89.2%) | 6,950 (83.5%) |
| 1 | 1,159 (8.4%) | 1,034 (12.4%) |
| 2 | 251 (1.6%) | 236 (2.8%) |
| 3 | 83 (0.6%) | 77 (0.9%) |
| 4 | 28 (0.2%) | 27 (0.3%) |
| GOLD stage |  |  |
| 0 | 12,602 (89.2%) | 6,950 (83.5%) |
| 1 | 830 (6.6%) | 744 (8.9%) |
| 2 | 592 (3.7%) | 536 (6.4%) |
| 3 | 95 (0.5%) | 90 (1.1%) |
| 4 | 4 (<0.1%) | 4 (0.0%) |
| Breathlessness (mMRC ≥ 1) | 2,553 (29.0%) | 2,553 (30.7%) |
| Deaths | 1,100 (5.9%) | 1,022 (12.3%) |

Data are presented as frequency (population-weighted percentage among all, sample percentage in age >=40y) unless otherwise specified. Breathlessness data were available and analyzed for people aged ≥40 years. Data were weighted against the US population. *Abbreviations:* FEV_1_ = forced expired volume in one second; FVC = forced vital capacity; GOLD = Global Initiative for Chronic Obstructive Lung Disease; mMRC = modified Medical Research Council breathlessness scale; pred = predicted normal value; SD = standard deviation; STAR = Staging of Airflow Obstruction by Ratio.

**Table S3.** Breathlessness and mortality by each STAR and GOLD stage when splitting stages 3 and 4 into two separate entities despite low numbers in GOLD stage 4.

|  | **Breathlessness**  Relative risk ratio (95% CI) | | **Mortality**  Hazard ratio (95% CI) | |
| --- | --- | --- | --- | --- |
|  | **Crude** | **Adjusted*** | **Crude** | **Adjusted*** |
| **STAR** |  |  |  |  |
| 0 | 1 | 1 | 1 | 1 |
| 1 | 1.6 (1.2 – 2.0) | 1.8 (1.4 – 2.4) | 3.2 (2.6 – 3.9) | 1.4 (1.2 – 1.7) |
| 2 | 3.2 (2.2 – 4.8) | 4.1 (2.9 – 6.0) | 5.8 (4.2 – 8.0) | 2.0 (1.4 – 2.9) |
| 3 | 7.5 (3.9 – 14.3) | 10.1 (4.8 – 21.5) | 9.1 (5.2 – 15.8) | 3.4 (2.1 – 5.8) |
| 4 | 31.2 (9.6 – 101.2) | 51.5 (16.7 – 158.6) | 9.2 (5.8 – 14.6) | 3.7 (2.3 – 5.9) |
|  | Correctly classified = 70 % | Correctly classified = 71 % | C statistic = 0.61 | C statistic = 0.81 |
| **GOLD** |  |  |  |  |
| 0 | 1 | 1 | 1 | 1 |
| 1 | 1.1 (0.8 – 1.5) | 1.3 (1.0 – 1.8) | 2.8 (2.3 – 3.5) | 1.2 (0.9 – 1.5) |
| 2 | 4.3 (3.3 – 5.8) | 4.9 (3.6 – 6.6) | 5.0 (4.0 – 6.2) | 2.1 (1.7 – 2.6) |
| 3 | 8.4 (4.6 – 15.2) | 9.7 (4.7 – 19.8) | 13.4 (8.2 – 21.9) | 5.5 (3.6 – 8.4) |
| 4 | 4.9 (0.5 – 48.4) | 10.5 (1.2 – 87.9) | 2.3 (0.3 – 21.4) | 1.3 (0.1 – 12.8) |
|  | Correctly classified = 70% | Correctly classified = 71% | C statistic = 0.61 | C statistic = 0.81 |

* Adjusted for age, sex, and body mass index.

*Abbreviations:* CI = confidence interval; GOLD = Global Initiative for Chronic Obstructive Lung Disease; STAR = Staging of Airflow Obstruction by Ratio.

**Table S4.** Prediction of breathlessness and mortality using FEV_1_/FVC or FEV_1_%pred in men and women.

|  | **Men** | **Women** |
| --- | --- | --- |
| **N** | 7,093 (50%) | 7,030 (50%) |
| **Breathlessness,** % correctly classified |  |  |
| FEV_1_/FVC | 75% | 67% |
| FEV_1_%pred | 75% | 68% |
| **Mortality,** C statistic (95% confidence interval) |  |  |
| FEV_1_/FVC | 0.81 (0.79 – 0.82) | 0.80 (0.79 – 0.82) |
| FEV_1_%pred | 0.81 (0.80 – 0.83) | 0.81 (0.80 – 0.83) |

Breathlessness and mortality were analyzed using multinomial logistic regression and Cox regression, respectively. FEV_1_/FVC or FEV_1_%pred are included as continuous variables in the models. All models were adjusted for age and body mass index.

**Table S5.** Breathlessness and mortality by STAR and GOLD stage with non-obstructive (stage 0) divided into two groups by presence or absence of Preserved Ratio Impaired Spirometry (PRISm).

|  | **Breathlessness**  Relative risk ratio (95% CI) | | **Mortality**  Hazard ratio (95% CI) | |
| --- | --- | --- | --- | --- |
|  | **Crude** | **Adjusted*** | **Crude** | **Adjusted*** |
| **STAR** |  |  |  |  |
| 0, without PRISm | 1 | 1 | 1 | 1 |
| 0, with PRISm | 1.9 (1.4 – 2.6) | 1.3 (0.9 – 1.9) | 3.6 (2.6 – 4.9) | 2.7 (2.1 – 3.6) |
| 1 | 1.6 (1.2 – 2.1) | 1.9 (1.4 – 2.4) | 3.5 (2.9 – 4.3) | 1.5 (1.2 – 1.9) |
| 2 | 3.3 (2.3 – 4.9) | 4.2 (2.9 – 6.1) | 6.3 (4.6 – 8.7) | 2.2 (1.5 – 3.1) |
| 3-4 | 9.2 (5.0 – 17.0) | 12.6 (6.3 – 25.3) | 9.8 (6.3 – 15.4) | 3.7 (2.4 – 5.7) |
|  | Correctly classified = 70% | Correctly classified = 71% | C statistic = 0.63 (0.62 – 0.65) | C statistic = 0.81 (0.80 – 0.82) |
| **GOLD** |  |  |  |  |
| 0, without PRISm | 1 | 1 | 1 | 1 |
| 0, with PRISm | 1.9 (1.4 – 2.6) | 1.3 (1.0 – 1.9) | 3.6 (2.6 – 4.9) | 2.8 (2.1 – 3.7) |
| 1 | 1.1 (0.8 – 1.5) | 1.4 (1.0 – 1.8) | 3.1 (2.5 – 3.8) | 1.2 (1.0 – 1.5) |
| 2 | 4.5 (3.4 – 5.9) | 4.9 (3.7 – 6.6) | 5.4 (4.3 – 6.7) | 2.2 (1.8 – 2.8) |
| 3-4 | 8.4 (4.7 – 14.9) | 9.8 (4.9 – 19.4) | 13.9 (8.6 – 22.6) | 5.7 (3.7 – 8.8) |
|  | Correctly classified = 70% | Correctly classified = 71% | C statistic = 0.63 (0.62 – 0.65) | C statistic = 0.81 (0.80 – 0.83) |

* Adjusted for age, sex, and body mass index.

*Abbreviations:* CI = confidence interval; GOLD = Global Initiative for Chronic Obstructive Lung Disease; STAR = Staging of Airflow Obstruction by Ratio.

PRISm= FEV1/FVC≥0.70 and FEV1<lower limit of normal.
